# Supplementary material for: Preparation, Characterization and Evaluation of the Anti-Inflammatory Activity of Epichlorohydrin-β-Cyclodextrin/Curcumin Binary Systems Embedded in a Pluronic®/Hyaluronate Hydrogel
Source: Int J Mol Sci. 2021 Dec 17;22(24):13566. doi: 10.3390/ijms222413566 (PMC8709285; doi:10.3390/ijms222413566)
Supplement: Supplementary file 1 [file ijms-22-13566-s001.zip › ijms-1445569-supplementary.pdf]

Preparation, characterization and evaluation of the anti-inflammatory activity of epichlorohydrin-β-cyclodextrin/curcumin binary systems embedded in a Pluron-ic®/hyaluronate hydrogel

Ana-María Fernández-Romero[1], Francesca Maestrelli[2], Sara García-Gil[3], Elena Talero[3], Paola Mura[2], Antonio María Rabasco[1] and María Luisa González-Rodríguez[1]*

1. Department of Pharmacy and Pharmaceutical Technology, Faculty of Pharmacy, Universidad de Sevilla, C/Prof. García González 2, 41012 Seville, Spain; anaferrom2@alum.us.es (AMFR); amra@us.es (AMR); malugoro@us.es (MLGR)

2. Department of Chemistry "Ugo Schiff" (DICUS), University of Florence, via Schiff 6, Sesto Fiorentino, 50019 Florence, Italy; francesca.maestrelli@unifi.it (FM); paola.mura@unifi.it (PM)

3. Department of Pharmacology, Faculty of Pharmacy, Universidad de Sevilla, C/ Prof. García González 2, 41012 Seville, Spain; saragarciagil2307@gmail.com (SGG); etalero@us.es (ET)

Correspondence:

*María Luisa González-Rodríguez (corresponding author): malugoro@us.es. Address Department of Pharmacy and Pharmaceutical Technology, Faculty of Pharmacy, Universidad de Sevil-la, C/Prof. García González 2, 41012 Seville, Spain

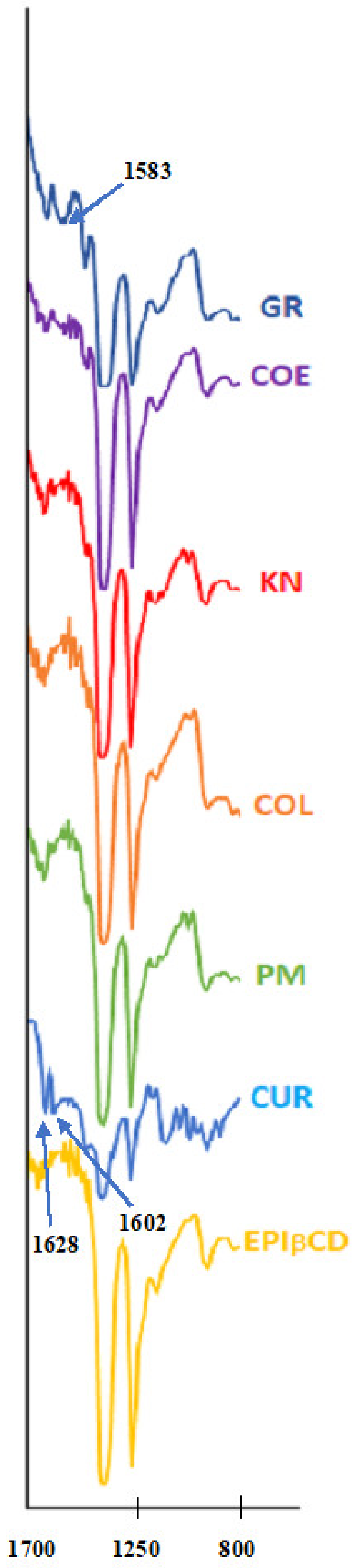

Figure S1: Augmented section of FTIR spectra. Abbreviations: GR, co-grinding; COE, co-evaporation; KN, kneading; COL, co-lyophilization.

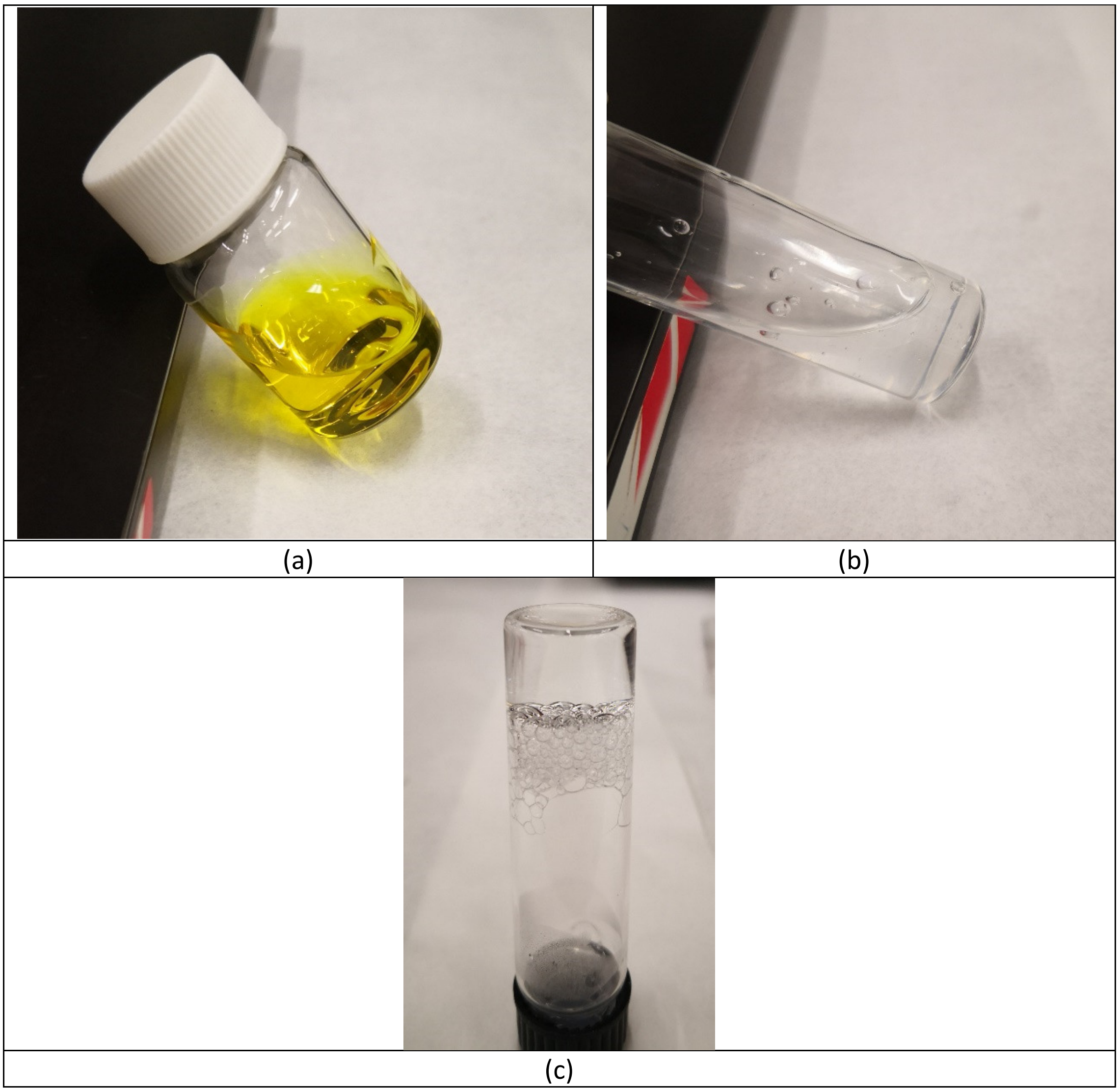

Figure S2: Photos of optimized hydrogel with CurEpi binary systema (a), optimized hydrogel empty (b) both heated at 20°C for 5 min and Pluronic® F-127 17% w/v (c) heated at 40°C for 5 min.
